# Laboratory Evaluation of Peripheral Blood Involvement in Mycosis Fungoides and Sézary Syndrome: Evolution of Flow Cytometry and Morphology Quantification and Interpretation

**DOI:** 10.3390/cancers18030434

**Published:** 2026-01-29

**Authors:** Lucy Fu, Payton Trimark, Yijie Liu, Hamza Tariq, Qing Chen, Yi-Hua Chen, Juehua Gao, Barina Aqil, Joan Guitart, Kristy Wolniak

**Affiliations:** 1 Department of Pathology, Feinberg School of Medicine, Northwestern University, Northwestern Memorial Hospital, Chicago, IL 60611, USA; yijie-liu@northwestern.edu (Y.L.); hamza.tariq@northwestern.edu (H.T.); qing.chen@northwestern.edu (Q.C.); y-chen5@northwestern.edu (Y.-H.C.); j-gao@northwestern.edu (J.G.); barina.aqil@northwestern.edu (B.A.); k-wolniak@northwestern.edu (K.W.); 2 Feinberg School of Medicine, Northwestern University, Chicago, IL 60611, USA; payton.fors@northwestern.edu; 3 Department of Dermatology, Feinberg School of Medicine, Northwestern University, Northwestern Memorial Hospital, Chicago, IL 60611, USA; j-guitart@northwestern.edu

**Keywords:** mycosis fungoides, Sézary syndrome, flow cytometry, Sézary cell morphology, TRBC1, blood staging, cutaneous T-cell lymphoma, laboratory optimization

## Abstract

**Background/Objectives**: Mycosis fungoides (MF) and Sézary syndrome (SS) are cutaneous T-cell lymphomas (CTCLs) with variable clinical outcomes. Peripheral blood (PB) involvement in MF/SS is an independent predictor of prognosis. Accurate laboratory determination of PB involvement by MF/SS cells, however, is an ongoing challenge. Both flow cytometry (FC) and morphology-based quantification are limited by the overlap of CTCL cells and reactive T-cells. This study looks at the optimization over time of CTCL blood burden evaluation. **Methods:** This retrospective study reviews CTCL blood assessment at Northwestern Memorial Hospital from 2012 to 2021. Test ordering and reporting practices for morphology-based Sézary cell counts and FC were evaluated. For each assay, quantitative and qualitative results were analyzed and compared including percentages and absolute counts of abnormal T-cell populations and pathologist interpretations. **Results:** A total of 514 patients were evaluated, with increasing numbers of both tests ordered over time. FC quantitative metrics showed a moderate to high correlation with morphology metrics, especially for absolute CD4+/CD7− counts (correlation coefficient = 0.901, *p*-value < 0.001). Qualitative pathologist interpretations had moderate agreement between methods (kappa = 0.58). The recent addition of TRBC1 clonality assessment to our FC assay further optimizes the evaluation for CTCL blood burden. **Conclusions:** Flow cytometry offers a reliable approach for blood staging in MF/SS, and morphologic assessment may be redundant. This study provides a foundation for designing a new FC approach with TRBC1. This comprehensive review of the evolution of our laboratory practices may serve as a guide for other institutions with similar clinical needs.

## Simple Summary

This study reviews how laboratory tests for blood involvement in mycosis fungoides (MF) and Sézary syndrome (SS), types of cutaneous T-cell lymphoma, have evolved at Northwestern Memorial Hospital. Two main methods are compared: flow cytometry and morphology-based cell counts. Over more than a decade, the number of tests ordered increased significantly, and flow cytometry became the preferred approach. Flow cytometry results strongly matched those from morphology, particularly for certain abnormal T-cell subsets. The recent addition of TRBC1 clonality testing in flow cytometry made it easier to confirm whether abnormal cells were truly cancerous, helping clarify cases that were previously uncertain. The findings suggest that flow cytometry, especially with TRBC1, is efficient and reliable for staging MF/SS, and that traditional morphology tests may no longer be necessary. This study proposes updating clinical guidelines to incorporate TRBC1, which could help physicians manage patients more effectively.

## 1. Introduction

Mycosis fungoides (MF) and Sézary syndrome (SS) are cutaneous T-cell lymphomas (CTCLs) with overlapping morphologic and phenotypic features; however, they show distinct disease courses and survival. MF is the most common CTCL subtype and accounts for more than half of all CTCL cases. Disease is generally indolent and limited to the skin, with variable distribution. Years, and sometimes decades, may pass from the development of early patches to more infiltrative plaques and solid tumors. Diagnosis is based on clinical presentation and histologic, immunophenotypic, and molecular evaluation of skin biopsies. Dissemination to the lymph nodes, solid organs, peripheral blood, and bone marrow may occur in advanced stages. Sézary syndrome (SS) is an aggressive form of CTCL which includes the triad of erythroderma, generalized lymphadenopathy, and detection of clonal neoplastic T-cells in skin, lymph nodes, and peripheral blood [1,2,3,4,5].

Neoplastic T-cells in both MF and SS show characteristic cerebriform nuclei (Sézary cells) and are typically CD4+/CD8− with loss of either CD7 and/or CD26 [1,2,3,4,5]. Due to the overlapping morphology and immunophenotype, advanced MF may be indistinguishable from SS; therefore, the two can be conceptualized as part of the same disease spectrum [1,2,3,4,5]. In fact, the International Society for Cutaneous Lymphomas (ISCL) and the European Organization for Research and Treatment of Cancer (EORTC) proposed a single revised staging system in 2007 to better stratify patients for prognostic and treatment determination. The ISCL/EORTC MF/SS staging system includes tumor (T), node (N), metastasis (M), and blood (B) classification to inform the overall clinical stage. Stage I and II disease is typically managed with skin-directed therapies. Advanced stage III and IV disease requires systemic approaches including chemotherapy, immune-therapy, and allogeneic stem-cell transplantation [5,6,7,8].

Determination of blood involvement and staging has particular significance in MF/SS, whereas blood classification is not used for other cutaneous lymphomas. Agar et al. showed that significant blood involvement in MF/SS has prognostic value independent of cutaneous and nodal involvement. Therefore, accurate determination of peripheral blood involvement by MF/SS cells is critical to management of these patients. The 2007 ISCL/EORTC MF/SS blood staging guidelines were based on quantifying Sézary cells (by morphology) or phenotypically atypical cells (by flow cytometry). B0 was defined as ≤5% (or <250/μL) Sézary cells by morphology or <15% CD4+/CD26−, CD4+/CD7−, or CD4+/CD26−/CD7− T-cells by flow cytometry. B2 was defined as positive clonal gene rearrangement with ≥1000/μL Sézary cells by morphology or CD4:CD8 ratio > 10, ≥40% CD4+/CD7− cells, ≥30% CD4+/CD26− cells, or >1000/μL CD4+/CD7− or CD4+/CD26− cells by flow cytometry. Stage B1 was defined as those not meeting either B0 or B2 criteria. Subsequently, the 2022 updates to the ISCL/EORTC MF/SS blood staging guidelines eliminated the percentage criteria for B0 as well as the clonality requirement and percentage criteria for B2 [9,10,11].

Northwestern Memorial Hospital (NMH) treats a large volume of patients with MF and SS. Following encounters for diagnosis, referral, and follow-up, a high number of analyses for blood involvement are ordered for disease monitoring. Due to the inherent challenges of identifying and accurately quantifying Sézary cells, our pathology and dermatology departments have developed and optimized blood burden reporting. In 2017, the flow cytometry laboratory underwent a quality improvement project to standardize our reporting, and these practices were subsequently included as published consensus guidelines [12,13]. Our comprehensive approach has evolved alongside the laboratory and clinical guidelines and includes quantitative and qualitative evaluations by both flow cytometric analysis and morphology review. In the current version of our assays, TRBC1 analysis for clonality further enhances more accurate determination of blood involvement and staging [14].

Limited studies have compared various laboratory methods in small cohorts or focused on the value of flow cytometry in clinical management [15,16,17,18,19,20,21,22,23,24]. To our knowledge, no work has been done in a large cohort to investigate the utility of the two main laboratory methods used to determined blood involvement in MF/SS—flow cytometry and morphology. With this study, we describe the evolution of assays over ten years at our high-volume tertiary referral center for CTCL, with particular attention to optimization of flow cytometric analysis. We share in detail our current flow cytometry and morphology-based evaluations, both from a laboratory performance perspective and a pathologist reporting perspective. Finally, we compare our current modalities and suggest improvements to our practice as it relates to relevant clinical parameters. We hope the results will influence laboratory and pathology practices to communicate the most meaningful and relevant data to our clinical colleagues.

## 2. Materials and Methods

### 2.1. Participants

This study was approved by the Northwestern University Institutional Review Board Committee. The Northwestern Medicine Enterprise Data Warehouse (EDW) was used for chart review and associated data collection. All patients evaluated and followed for CTCL with peripheral blood evaluations at NMH between 2012 and 2021 (including laboratory sample preparation, assay performance, and reporting practices) were reviewed in detail. Flow cytometric analyses and morphology-based Sézary cell counts were included, with particular attention to the standardization of flow cytometry practices.

### 2.2. Measures

#### 2.2.1. Morphology Review (Sézary Cell Count)

At least 4 mL of whole blood was collected in an EDTA anticoagulated tube. Six smears were manually prepared. Three smears were then hand-stained with a modified Wright-Giemsa protocol (decreased time in Wright-Giemsa and buffer solutions, Figure S1). Smears were then evaluated for suspected Sézary cells with homogeneous, smooth/dense nuclear chromatin pattern and evidence of nuclear folding. A 200-cell manual differential was performed on each of the 3 stained slides for a total 600-cell differential. The number of lymphocytes with convoluted nuclei was tallied separately from normal lymphocytes as part of the differential. The Sézary cell count worksheet includes the concurrent CBC, total number of cells counted (should be 600, but may be less in cytopenic patients), and percentages and calculated absolute counts per μL.

**Figure 1:**
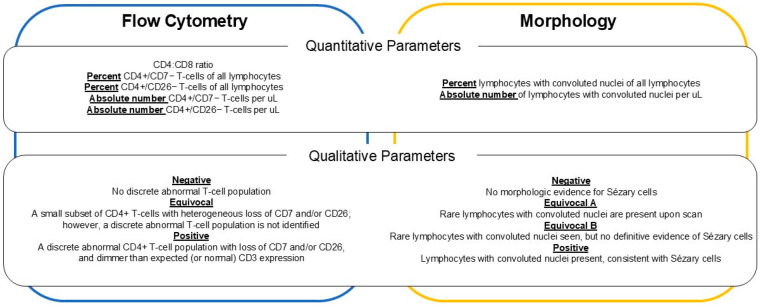
Quantitative parameters and qualitative interpretations reported by flow cytometry and by morphology.

Quantitative parameters reported in morphology review include percent of lymphocytes with convoluted nuclei out of total lymphocytes and absolute number of lymphocytes with convoluted nuclei per μL. Qualitative interpretations by the pathologist were categorized as Negative, Equivocal (A and B), or Positive as shown in Figure 1.

#### 2.2.2. Flow Cytometric Analysis (Sézary Panel)

Peripheral blood samples were collected in heparin and processed within 24 h. Following red blood cell lysis with ammonium chloride, the cells were incubated with antibodies at 40 °C for 15 min followed by one wash. To assess for CTCL T-cells in the peripheral blood, we performed 6-color or 8-color flow cytometric analysis. Data collection was performed on a Becton Dickinson Biosciences (BD, Franklin Lakes, NJ, USA) LSR II flow cytometer employing 488 nm and 635 nm excitation and using 505 nm longpass (LP), 550 nm dichroic LP (DCLP), 530 nm bandpass (BP), 575 nm BP, 685 nm DCLP, 695 nm BP, 735 nm DCLP, and 780 nm BP for the 488 nm excitation source, and 735 nm DCLP, 660 nm BP, and 780 nm BP for the 635 nm excitation source. Data analysis was performed using FACSDiva software version 9.0 (BD) and Kaluza software version 2.1 (Beckman Coulter, Indianapolis, IN, USA). The antibodies used to evaluate for patterns of abnormal T-cell antigen expression are listed in Table S1.

The FCS files from flow cytometry cases analyzed prior to 2017 were reanalyzed in Kaluza (BC) using the updated analysis template to generate the parameters.

Quantitative parameters by flow cytometry include: CD4:CD8 ratio, percent CD4+/CD7− T-cells of all lymphocytes, percent CD4+/CD26− T-cells of all lymphocytes, absolute number of CD4+/CD7− T-cells per μL, and absolute number of CD4+/CD26− T-cells per μL. Qualitative interpretations by the pathologist were categorized as Negative, Equivocal, or Positive as shown in Figure 1.

### 2.3. Statistical Analysis

Descriptive statistics for our patient cohort, flow quantitative and qualitative results, and morphology quantitative and qualitative results were performed. A one-sided *t*-test with FDR-adjusted *p*-value was used to compare quantitative measures between qualitative interpretation categories for both flow and morphology results. The Pearson correlation coefficient with FDR-adjusted *p*-value was calculated to compare flow quantitative measures with morphology quantitative measures. The Cohen weighted kappa correlation coefficient was calculated to compare flow qualitative interpretation categories with morphology qualitative interpretation categories.

## 3. Results

### 3.1. CTCL Patients and Ordering Practices for Peripheral Blood Evaluations Between 2012 and 2021

Between 2012 and 2021, our laboratory tested samples from 514 patients undergoing evaluation for CTCL (Table 1). In our patient cohort there was a slight male predominance (53% vs. 47%). The median age of patients was 61 years old, and the range was from 48 to 71 years old. The majority of patients were White/Caucasian (60%) followed by Black/African American (14%). At time of data analysis, 36 patients (7%) had undergone a bone marrow transplant, and 49 patients (9.5%) were deceased per the electronic medical record.

**Figure 2:**
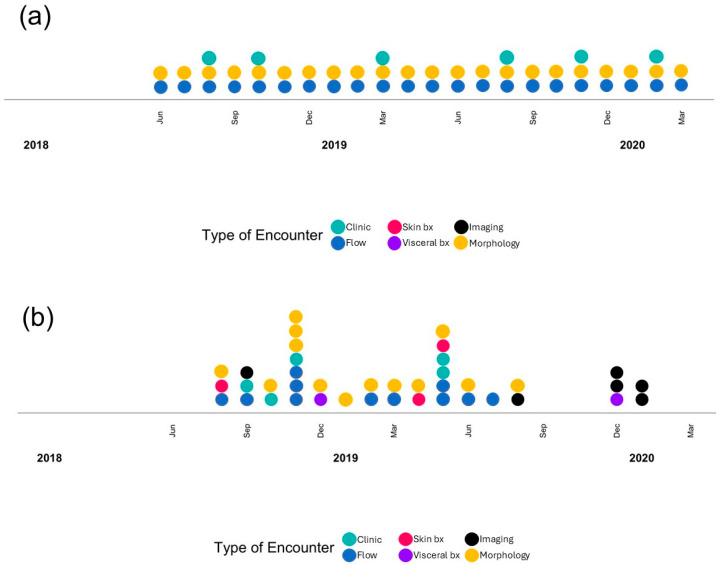
Example of test ordering practices for selected CTCL patients: patient (**a**) with stable disease and patient (**b**) with more aggressive clinical course. Each column corresponds to an encounter, mostly outpatient for patient (**a**) and both outpatient and inpatient for patient (**b**).

CTCL patients follow-up periodically with the dermatology clinic. At initial and follow-up encounters, flow cytometry, morphology review, skin biopsy, imaging, and visceral biopsy can be ordered for evaluation. To demonstrate the overall evaluation approach for CTCL at NMH, Figure 2 illustrates the timing and frequency of laboratory evaluations ordered at each visit for two representative patients. Patient (a) has stable disease and returns for monthly follow-ups to monitor management outcomes. Flow cytometry and morphology evaluation are ordered at each encounter and do not show significant involvement or changes over time. Patient (b) has rapidly progressive disease with profound peripheral blood and visceral involvement. Patient (b) is brought back to the clinic multiple times per month with accompanying laboratory studies ordered at each encounter. Additional flow cytometry and morphology evaluations are ordered between visits for closer monitoring.

**Figure 3:**
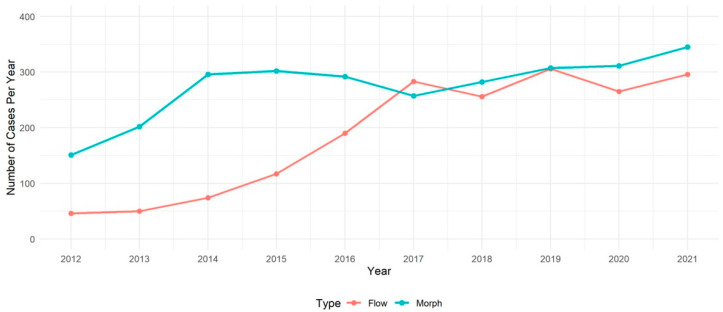
Flow and morphology cases ordered for evaluation and monitoring of CTCL patients at Northwestern Memorial Hospital from 2012 to 2021. Over ten years, morphology cases ordered continued to outnumber flow cytometry cases ordered. However, the difference in 2021 was by a much narrower margin, reflecting the increased use of flow cytometry.

The total numbers of flow cytometry and morphology evaluations ordered for CTCL patients increased between 2012 and 2021 (Figure 3). The number of flow cytometry cases ordered grew rapidly between 2012 and 2017 before more stabilized growth (from 46 up to 296), and the number of morphology cases ordered grew rapidly between 2012 and 2014 before showing more steady growth (from 151 up to 345). In 2012, more than three times as many morphology evaluations were ordered compared to flow (151 vs. 46). By 2021, the number of morphology evaluations still outpaced flow, but by a narrower margin (345 vs. 296).

### 3.2. Morphology Evaluation of Sézary Cells

Microscopic evaluation of peripheral blood for Sézary cells is unique to very few institutions. In a 2020 survey, only two of ten academic centers with expertise in CTCL routinely provide this service [25]. At NMH, this assay consists of a time-intensive manual differential of 600 cells across three peripheral blood smear slides. A concurrent CBC with differential is incorporated into the final report to calculate the percentage of lymphocytes with convoluted nuclei (of all lymphocytes) and estimated absolute number of lymphocytes with convoluted nuclei per μL. As lymphocytes with convoluted nuclei occur in normal, reactive, and neoplastic settings, a pathologist additionally reviews the peripheral blood smear and renders a qualitative interpretation of peripheral blood involvement (Figure 1).

**Figure 4:**
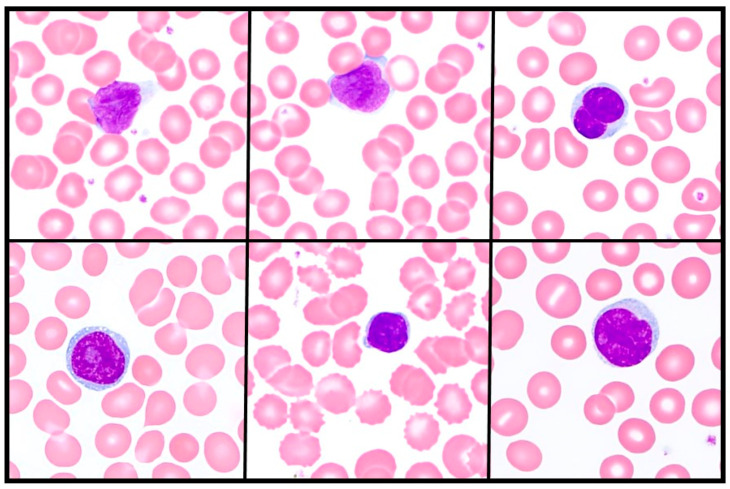
Spectrum of Sézary cell morphology (600× magnification). Textbook features of Sézary cells include a homogeneous, smooth/dense nuclear chromatin pattern in addition to nuclear folding. In practice, Sézary cells can show a spectrum of nuclear and cytoplasmic morphology, requiring time-intensive training for morphology review.

Specific technical requirements and extensive training are necessary before onboarding this assay. Peripheral blood smears must be well-prepared and hand-stained with Wright-Giemsa to optimize nuclear features. The staining protocol is optimized for this assay to allow visualization of the convoluted nuclei, including less time in stain and buffer solutions. Accurate and reproducible 600-cell counts require up to 20–60 min per case, compared to 3–5 min for a standard 100-cell differential count. Laboratory technicians are extensively trained on recognizing features of Sézary cells including homogeneous, smooth/dense nuclear chromatin pattern, in addition to nuclear folding. In practice, Sézary cells can show a spectrum of nuclear and cytoplasmic morphology (Figure 4). Performers of this assay must show proficiency with accurate evaluation of 20 active cases. The entire training period ranges from 8 to 16 weeks depending on other duties. At the time of data analysis, only five technicians have been trained in the previous 45 years to evaluate/count Sézary cells. Two technicians perform 10–20 cases/week.

### 3.3. Evolution of Flow Cytometric Analysis in CTCL

**Figure 5:**
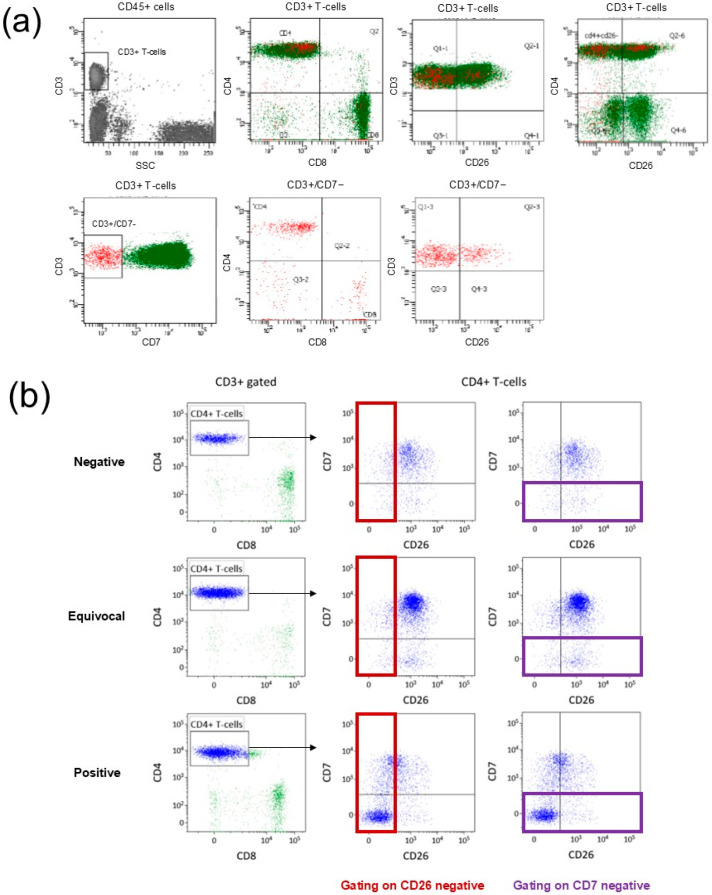
Flow analysis and gating strategies (**a**) prior to 2017 and (**b**) after 2017, standardized to align with staging guidelines. Prior to 2017, numerous gating strategies and varied interpretations between pathologists were difficult to interpret. In 2017, a simplified and more effective gating strategy was adopted which clearly highlights CD4+ T-cell populations with loss of CD7 and/or CD26.

Flow cytometric analysis began as an adjunct to the morphology Sézary cell count, but over the years became the recommended diagnostic approach [12,13,14,16,18,21,22,23,26]. Since 2012, the basic backbone of the flow Sézary panel in our laboratory has remained stable and includes CD3, CD4, CD8, CD7, and CD26. In addition to the quantitative parameters reported by flow, our pathologists also render qualitative interpretations, which have evolved over time. In general, assays for neoplastic T-cells are more challenging than evaluation for atypical monotypic B-cell populations, particularly in the era before TRBC1 clonality assessment at our institution. From 2012 to 2017, numerous gating strategies were used incorporating CD7 and CD26 (Figure 5a). Gating strategies and interpretations varied over time, and interpretations varied widely between pathologists (example report in Figure S2).

From the clinician’s perspective, the array of numbers and percentages reported were difficult to interpret. Reflecting ongoing efforts to improve pathologist–clinician communication [27,28], our hematopathologists and dermatologists determined that flow cytometric MF/SS evaluation reports should include presence or absence of abnormal T-cells, phenotype of abnormal cells, and quantity of abnormal cells for disease burden and staging determination. In 2017, a new standardized reporting methodology was implemented as a quality improvement project in our flow cytometry laboratory.

In alignment with the subsequently published consensus guidelines, the four key elements included in the updated flow cytometric analysis included (1) six antibodies: CD45, CD3, CD4, CD8, CD7, and CD26; (2) a reliable analysis template to detect abnormal T-cells (uncharacteristic phenotypes may lack staining for CD3 or CD45); (3) gating strategies based on identifying distinct homogeneous populations phenotypically different from expected normal T-cells; and (4) estimated blood concentration of abnormal cells calculated by a dual-platform method [12,13].

Figure 5b shows the simplified and more effective gating strategy which clearly highlights CD4+ T-cell populations with loss of CD7 and/or CD26. The “Positive” case shows a distinct homogeneous (“discrete”) population when applying the updated gating strategy. In the updated report, percentages of phenotypically abnormal T-cell populations were reported in standardized table format along with calculated estimated absolute counts based on concurrent white blood cell count (WBC) from CBC (Figure 1, example report in Figure S3).

To bring uniformity to the report, the pathologist also rendered a qualitative interpretation of “discreteness” of the atypical T-cell population with one of three standardized comments (Figure 1). These additional qualitative descriptors signaled to the clinician our best interpretation of true peripheral blood involvement and helped to overcome the challenge of communicating positive blood involvement in patients with lymphopenia and relatively low quantitative measures [29].

### 3.4. Correlation of Quantitative Parameters and Qualitative Interpretations

**Figure 6:**
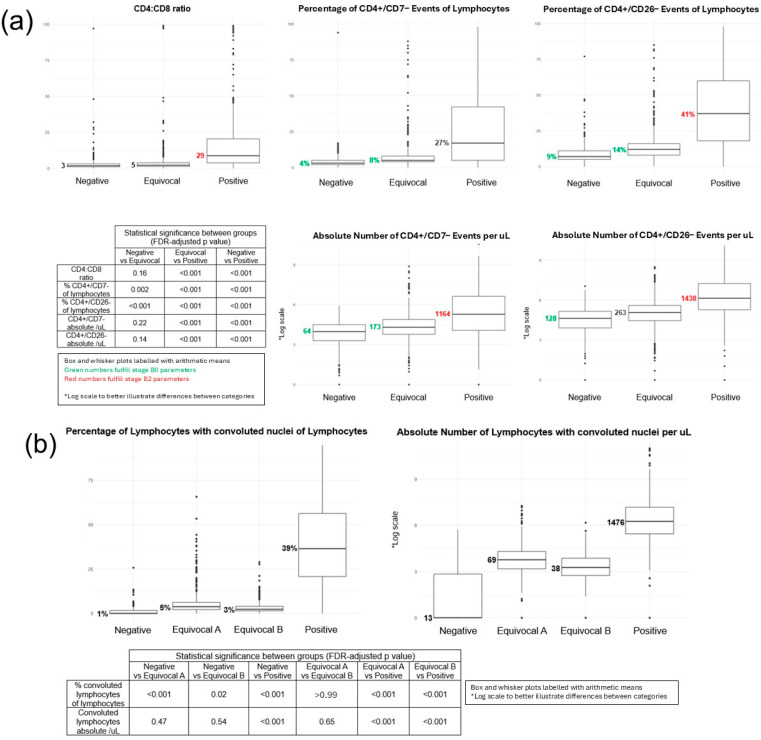
Correlation of quantitative measures and qualitative interpretation categories (**a**) by flow cytometry and (**b**) by morphology: (**a**) shows that flow cases with Positive qualitative interpretations had higher quantitative measures when compared to Equivocal and Negative cases; (**b**) shows that morphology cases with Positive qualitative interpretations correlated to higher quantitative measures when compared to Equivocal A, Equivocal B, and Negative cases. Overall, Equivocal and Negative cases did not consistently correlate with significantly different quantitative measures by either flow or morphology.

As our standard reporting approach includes both qualitative and quantitative elements, we compared the parameters reported for each methodology. Flow cytometry quantitative measures between different flow cytometry qualitative interpretation categories (Negative, Equivocal, and Positive) are shown in Figure 6a. The mean of quantitative measures for Positive was significantly higher than those of both Negative and Equivocal (*p*-values < 0.001). The mean of quantitative measures for Negative was less than the mean of quantitative measures for Equivocal (however not all differences were significant).

Morphology quantitative measures between different qualitative morphology interpretations (Negative, Equivocal A, Equivocal B, and Positive) are shown in Figure 6b. The mean of quantitative measures for Positive was significantly higher than the means of all Negative, Equivocal A, and Equivocal B (*p*-values < 0.001). The mean of quantitative measures for Negative was slightly less than the mean of quantitative measures for both Equivocal A and Equivocal B; however, the differences were not significant between any of the three categories.

### 3.5. Correlation Between Flow Cytometry and Morphology Reporting

**Figure 7:**
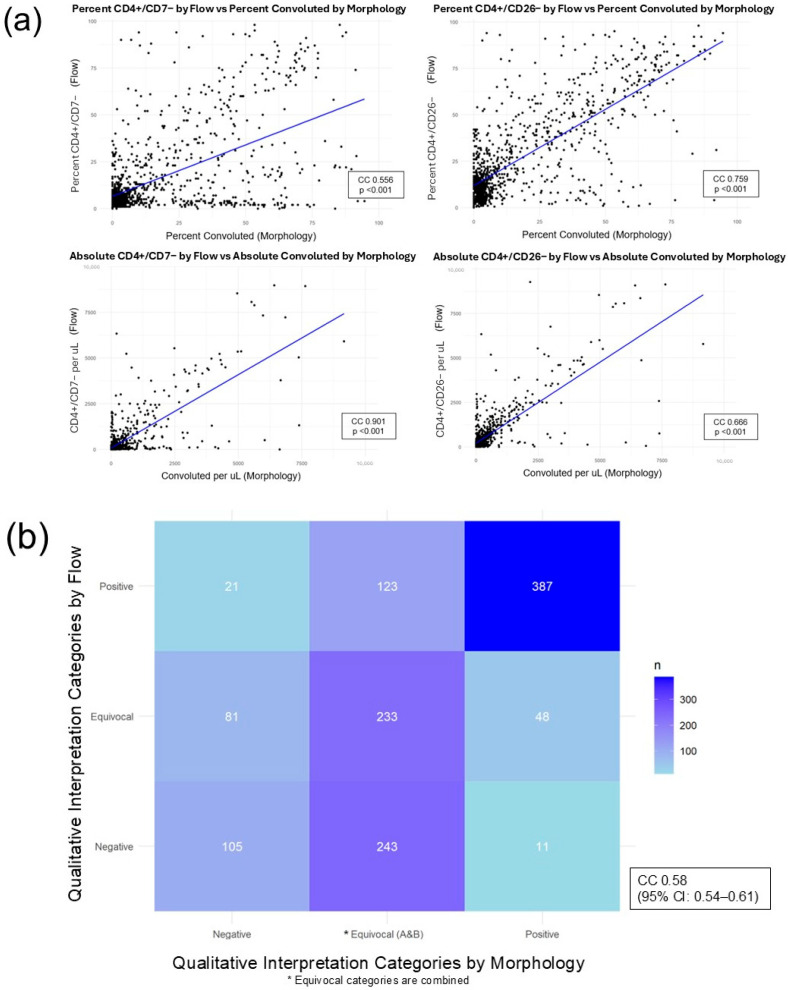
Comparing flow vs. morphology reporting (**a**) when considering quantitative measures and (**b**) qualitative interpretations: (**a**) shows that quantitative measures by flow vs. by morphology have moderate to high correlation; (**b**) shows that qualitative interpretations by the pathologist in flow vs. in morphology have moderate agreement.

As one of the few institutions that performs both flow cytometry and morphology assessments for MF/SS in the peripheral blood, we compared all cases with paired flow cytometry and morphology reviews. Quantitative measures by flow cytometry and by morphology for matched encounters are plotted in Figure 7a. There is overall moderate to high correlation between reported percentages and absolute counts of abnormal lymphocytes between the two assays (correlation coefficients ≥ 0.556, *p*-values < 0.001). When considering absolute counts, CD4+/CD7− phenotype cells identified by flow had the higher correlation with Sézary cells identified by morphology (correlation coefficient = 0.901, *p*-value < 0.001). However, when considering percentage of atypical cells, CD4+/CD26− phenotype cells identified by flow had the higher correlation with percentage Sézary cells identified by morphology (correlation coefficient = 0.759, *p*-value < 0.001).

When comparing pathologists’ qualitative interpretation of blood involvement by flow cytometry vs. by morphology, the weighted Cohen’s kappa correlation coefficient was 0.58 (95% CI: 0.54–0.61), indicating moderate agreement (Figure 7b). Out of 531 cases with a discrete Positive population by flow cytometry, 123 (23%) were interpreted as Equivocal by morphology review, and 21 (4%) were interpreted as Negative by morphology review. Out of 446 cases that were Positive by morphology review, 48 (11%) were Equivocal by flow cytometry, and 11 (2%) were Negative by flow cytometry.

### 3.6. Ongoing Optimization of the CTCL Flow Cytometry Panel

Evidence of T-cell receptor (TCR) clonality has been a component of different MF/SS blood staging criteria [7,11]. Molecular PCR-based TCR gene rearrangement methods, however, may detect unrelated T-cell clones and are not uniformly ordered for evaluation. In 2023, our flow cytometry laboratory introduced T-cell receptor (TCR) β-chain constant region 1 (TRBC1) to T-cell assays including the Sézary panel. TRBC1 is one of two constant regions of TCR β found on alpha–beta T-cells. During TCR gene rearrangement, a cell irreversibly selects one of the two constant regions (TRBC1 or TRBC2) for expression in a random and mutually exclusive manner. Its interpretation in flow cytometry is akin to kappa and lambda analysis in B-cells. As established in our laboratory, expression of TRBC1 in either >85% or <15% of events in a cell population is consistent with evidence of clonality in that population.

**Figure 8:**
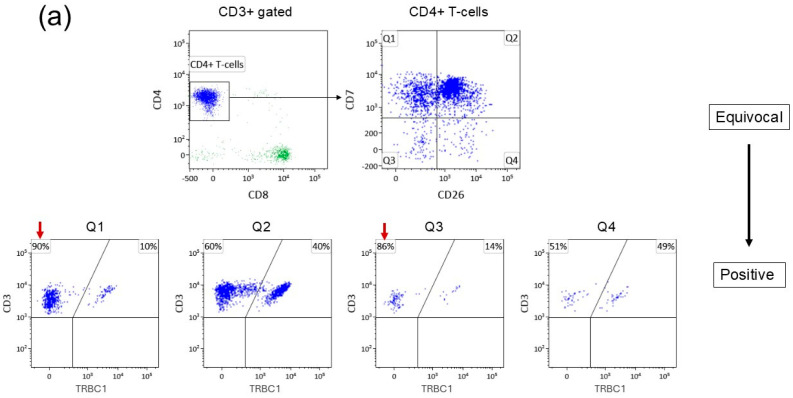

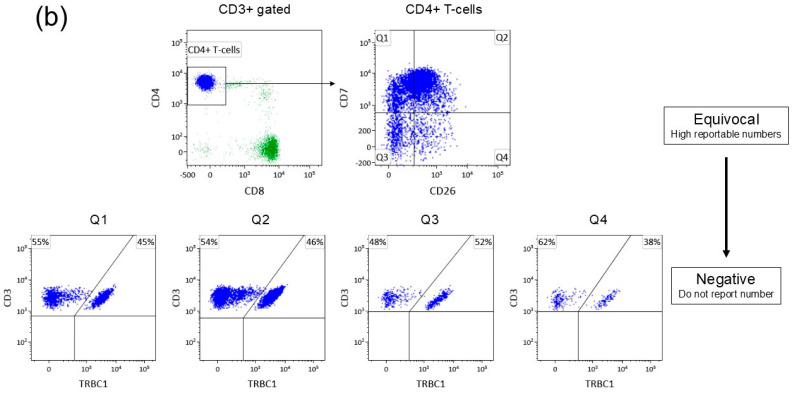
Addition of TRBC1 further refines quantitative measures and interpretation of disease involvement including recategorization from (**a**) Equivocal to Positive and (**b**) Equivocal to Negative. TRBC1 expression in either >85% or <15% of a population of CD4+ T-cells is interpreted as clonal/positive (red arrows).

Adoption of TRBC1 has further refined both quantitative and qualitative interpretations for blood involvement. Patients with cases previously reported as Equivocal are now shown to have cases definitively Positive for a clonal population at a low count (Figure 8a). Other patients with previous cases which were Equivocal but still reported large numbers of phenotypically atypical cells are now shown to have cases that are polyclonal and definitively Negative for blood involvement (Figure 8b).

## 4. Discussion

The clinical significance of blood staging in MF/SS is well established; however, the approach to determining blood involvement by neoplastic T-cells is an ongoing challenge for laboratories [9,10,23,24,25,29]. We developed an iterative multiparameter approach that provides both qualitative and quantitative outputs for peripheral blood morphology Sézary cell and flow cytometric analysis. In this study we retrospectively compared the different parameters assessed and reported at our institution over the span of 10 years. Our study shows that pathologists’ Positive qualitative interpretation in both flow cytometry and morphology assays correlated with a high quantitative level of disease involvement by both methods. Pathologists’ Negative or Equivocal interpretations correlated with a low quantitative level of disease involvement by both methods, and there was no significant difference between the Negative and Equivocal categories. In fact, based on the current staging guidelines, Negative and Equivocal categories both generally fulfill criteria for stage B0 (Figure 6a).

Morphology-based evaluations for Sézary cells have not significantly changed throughout the course of our study, and the general methodology is consistent even today. While the original staging guidelines relied on morphologic Sézary cell counts, the newer guidelines no longer require the morphologic counts [6,7,11]. When comparing quantitative measures between methods, the absolute number of Sézary cells identified by morphology shows a high correlation with phenotypically atypical cells identified by flow cytometry. Absolute CD4+/CD7− values showed higher correlation with Sézary cell counts than absolute CD4+/CD26− values. These findings suggest that flow cytometric identification of atypical cells with our current panel closely approximates that of morphology evaluation of Sézary cells.

The correlation between qualitative interpretation categories (Positive, Equivocal, and Negative) between morphology and flow cytometry methods is only moderate. This is not surprising since the differences between Equivocal and Negative categories are not significant within each assay, and any perceived difference in blood involvement by the pathologist is subjective. It is worth noting that the “hottest” correlations in the heatmap (Figure 7b) are when both the morphology and flow are clearly Positive.

Since a gold standard approach to identifying neoplastic T-cells in the peripheral blood is not available, the positive correlation between different methodologies provides support for the use of the current flow cytometry panel. Based on current capabilities as well as the widespread availability of flow cytometry, we suggest that morphology assays for Sézary cells may be redundant and could be eliminated from MF/SS blood staging evaluation, particularly since optimal training and evaluation by morphology is a time and labor-intensive assay.

In terms of pathologist-rendered interpretations, the Equivocal category was created for instances of diagnostic ambiguity. However, the findings in both flow cytometry and morphology data show that Equivocal and Negative categories did not consistently correlate with significantly different quantitative measures (Figure 6). We suggest the Equivocal category is essentially equivalent to the Negative category and propose that the qualitative categories could be streamlined to a two-tiered system of Positive and Negative with elimination of Equivocal category reporting altogether. Furthermore, both Negative and Equivocal categories correlate with low quantitative values, compatible with stage B0. If confirmed that low-level involvement portends no significant clinical significance, the blood staging guidelines could also be simplified to a binary system.

The addition of TRBC1 to our flow cytometry panel further supports this transition, and we suggest using evidence of clonality by TRBC1 as the true determinant of Positive rather than relying on more subjective interpretations based on “discreteness”. In fact, TRBC1 may be superior to molecular PCR-based methods in the evaluation of MF/SS blood involvement. Since TRBC1 allows for assessment of clonality in conjunction with immunophenotype, incorporation of this antibody allows for immediate determination of whether a subset of phenotypically atypical T-cells is truly clonal and neoplastic. This further eliminates inter-observer subjective interpretations in cases with low blood burden.

Although this study is a comprehensive review of ten years’ data at a high-volume CTCL referral center, a few limitations remain. This study is a retrospective review, and the scope of the current data does not fully address clinical outcomes and follow-up. Although earlier flow cytometry cases from 2012 to 2017 were reanalyzed, there may be inconsistencies in the quantification and qualitative interpretations when compared to data from only 2017 onwards. The brief discussion of TRBC1 for clonality assessment by flow is limited since the laboratory is currently validating use of TRBC2. This addition would further improve on clonality assessment by flow cytometry when compared to using TRBC1 alone [30]. The overall findings are generalizable to other institutions with similar assays; however, most laboratories do not offer morphology Sézary counts and may not immediately benefit from a subset of the results. Additional confirmation of our findings in morphology and comparing flow and morphology may not be possible or widespread. Furthermore, as a single-institution study, it is not possible to confirm that morphology training and subjective pathologist qualitative interpretations are consistent across different laboratories and practice settings. Despite these limitations, the overall results still support the movement in the CTCL community towards reliance on flow cytometry for purposes of blood stage determination and definitively eliminating the necessity for morphology review.

## 5. Conclusions

Current clinical management of MF/SS relies on accurate and reliable evaluation of blood staging. This comprehensive review of the evolution of our laboratory practices, including comparison of flow cytometry and morphology-based methods, may serve as a guide for other institutions with similar clinical needs. Further modifications to the ISCL/EORTC MF/SS blood staging criteria may be considered given our current flow cytometry capabilities including TRBC1. Additional investigation of TRBC1 in refining quantitative results of blood involvement is underway, including the clinical implications of potential restaging.

## Figures and Tables

**Table 1:** Demographics of CTCL patients evaluated at Northwestern Memorial Hospital from 2012 to 2021.

Characteristic	N = 514
Gender	
Female	243 (47.0%)
Male	271 (53.0%)
Race	
American Indian or Alaska Native	1 (0.2%)
Asian	19 (3.7%)
Asian Indian	1 (0.2%)
Black or African American	74 (14.0%)
Filipino	1 (0.2%)
Native Hawaiian or Other Pacific Islander	1 (0.2%)
None of the above	36 (7.0%)
Other Asian	2 (0.4%)
Unknown	69 (13.0%)
White	310 (60.0%)
Multiracial	7 (1.4%)
Ethnicity	
Hispanic	31 (6.0%)
Not Hispanic	404 (79.0%)
Unknown	79 (15.0%)
Deceased	49 (9.5%)
Transplant	36 (7.0%)

## Data Availability

Research data is available upon request to the corresponding author.

## Supplementary Materials

The following supporting information can be downloaded at: https://www.mdpi.com/article/10.3390/cancers18030434/s1, Figure S1: Modified peripheral blood staining protocol for Sézary cell count; Figure S2: Example of “Equivocal” flow report prior to standardization; Figure S3: Example of “Equivocal” flow report post-standardization; Table S1: Antibodies used in T-cell flow analysis.
