# Supplementary material for: Laboratory Evaluation of Peripheral Blood Involvement in Mycosis Fungoides and Sézary Syndrome: Evolution of Flow Cytometry and Morphology Quantification and Interpretation
Source: Cancers (Basel). 2026 Jan 29;18(3):434. doi: 10.3390/cancers18030434 (PMC12896628; doi:10.3390/cancers18030434)
Supplement: Supplementary file 1 [file cancers-18-00434-s001.zip › cancers-4043805-supplementary.pdf]

BECKMAN COULTER (BCI) TRU-COLOR WRIGHT STAINING GUIDELINES

VESSEL	CONTENTS	Timing PB / Sezary	Timing TP - Direct	Timing ASP	When to change / Notes
1	Absolute Methanol	2 mins	2 mins	2 mins	Pour fresh each morning.
2	1 bottle of BCI Wright Stain with 100ml of BCI Wright - Giemsa added to the bottle.	3 mins	4 mins	9 mins	Replace with fresh stain each Monday or if the staining appears weak.
3	BCI Wright/Giemsa stain -30cc<br>Sigma Buffer (pH7.2) -180cc	9 mins	12 mins	27 mins	• Made fresh for every batch of slides.<br>• Stain should be added ~1min prior to adding the slides.<br>• Halfway through the staining process, gently agitate the slide carrier.
4	DI water/ Methanol (150/50)	1 dip	2 dips	1 dip	Change in morning and afternoon.
5	DI water	10 dips	10 dips	10 dips	Change with every batch.
6	DI water	10 dips	10 dips	10 dips	Change with every batch.
7	DI water	10 dips	10 dips	10 dips	Change with every batch.
8	Wright Stain Dip Buffer pH 6.0	15 dips	15 dips	15 dips	Change if visibly appears blue.
9	DI Water	10 dips + 1 min	10 dips + 1 min	10 dips + 2 mins	Change with every batch.

- Note: The staining times may be adjusted slightly as necessary (section thickness, lot change, etc). Consult senior staff before any staining times are adjusted.

Figure S1. Modified peripheral blood staining protocol for Sézary cell count.

Test Performed

Flow Cytometry Immunophenotyping

Interpretation

Flow cytometric immunophenotypic analysis of the peripheral blood reveals a CD3+ CD7- T cell population that is predominantly CD4+ with partial loss of CD26 (see comment).

Comment: The population comprises 4% of the T cells and <1% of the total cells. The findings are not definitive for peripheral blood involvement by Sezary cells.

Immunophenotyping Results

Based on CD45 (LCA) staining and side scattered light (SS) characteristics, the red blood cell lysis prepared peripheral blood sample was found to contain

Approximately:
38% lymphocytes (bright CD45+, low SS intensity)
3% monocytes (bright CD45+, intermediate SS intensity)
53% granulocytes (dim CD45+, high SS intensity)

T cell characteristics (Selected based on CD3+ antigen expression)

CD7	Positive
CD4	60%
CD8	33%
CD4+/CD26-	26%

CD3+ CD7-: 4%

CD4+	69%
CD8+	13%
CD26	Partial

Figure S2. Example of “Equivocal” flow report prior to standardization.

Test Performed

Flow Cytometry Immunophenotyping

Interpretation

A small subset of CD4+ T cells show loss of CD26, however a discrete phenotypically abnormal T cell population is not identified.

Immunophenotyping Results

Concurrent WBC: 10.6 K/uL

	% of Total Cells	Absolute Count (per uL)
Lymphocytes	19%	2014
Monocyte	8%	
Granulocytes	72%	

Gated on Lymphocytes

	% of Lymphocytes	Absolute Count (per uL)
CD3+ T cells	68%	1370
CD19+ B cells	24%	483
CD56+/CD3- NK Cells	4%	81

CD3+CD4+	42%
CD3+CD8+	24%
Total CD4:CD8 ratio	1.8

CD3+/CD4+/CD7-	4%	76
CD3+/CD4+/CD26-	15%	297
CD3+/CD4+/CD7-/CD26-	3%	51

The absolute counts are estimates based on a CBC from the same day the specimen is received. Minor variations may be noted between the absolute counts from flow cytometric immunophenotypic analysis and the hematology analyzer due to instrument and population gating differences.

Figure S3. Example of “Equivocal” flow report post-standardization.

Antigen	Clone	Vendor
**CD3**	SK7	BD
**CD4**	13B8.2	BC
**CD7**	CD7-6B7	BIOLEGEND
**CD8**	SFCI21Thy2D3	BC
**CD25**	2A3	BD
**CD26**	4EL-1C7	BC
**CD45**	J33	BC
**CD52**	4C8	BD
**TRBC1**	JOVI.1	BIOLEGEND

Table S1. Antibodies used in T-cell flow analysis.
